# Natural Compounds and Aging: Between Autophagy and Inflammasome

**DOI:** 10.1155/2014/297293

**Published:** 2014-09-14

**Authors:** Shih-Yi Chuang, Chih-Hung Lin, Jia-You Fang

**Affiliations:** ^1^Pharmaceutics Laboratory, Graduate Institute of Natural Products, Chang Gung University, 259 Wen-Hwa 1st Road, Kweishan, Taoyuan 333, Taiwan; ^2^Research Center for Industry of Human Ecology, Chang Gung University of Science and Technology, Kweishan, Taoyuan 333, Taiwan; ^3^Center for General Education, Chang Gung University of Science and Technology, Kweishan, Taoyuan 333, Taiwan; ^4^Chronic Diseases and Health Promotion Research Center, Chang Gung University of Science and Technology, Kweishan, Taoyuan 333, Taiwan; ^5^Chinese Herbal Medicine Research Team, Healthy Aging Research Center, Chang Gung University, Kweishan, Taoyuan 333, Taiwan

## Abstract

Aging, a natural physiological process, is characterized by a progressive loss of physiological integrity. Loss of cellular homeostasis in the aging process results from different sources, including changes in genes, cell imbalance, and dysregulation of the host-defense systems. Innate immunity dysfunctions during aging are connected with several human pathologies, including metabolic disorders and cardiovascular diseases. Recent studies have clearly indicated that the decline in autophagic capacity that accompanies aging results in the accumulation of dysfunctional mitochondria, reactive oxygen species (ROS) production, and further process dysfunction of the NACHT, LRR, and PYD domains-containing protein 3 (NLRP3) inflammasome activation in the macrophages, which produce the proinflammatory cytokines. These factors impair cellular housekeeping and expose cells to higher risk in many age-related diseases, such as atherosclerosis and type 2 diabetes. In this review, we investigated the relationship between dysregulation of the inflammasome activation and perturbed autophagy with aging as well as the possible molecular mechanisms. We also summarized the natural compounds from food intake, which have potential to reduce the inflammasome activation and enhance autophagy and can further improve the age-related diseases discussed in this paper.

## 1. Introduction

Aging is a natural physiological process that affects a person with a progressive loss of physiological integrity over the passage of time. According to the World Health Organization (WHO), the proportion of the world's population over the age of 60 will rise to 22% by 2050 [1]. However, although everyone grows older as time passes, the degree of change of physiological function in different individuals may differ. Aging factors may have a variety of different sources, including changes in genes, cell imbalance, and organ senescence.  Table 1 summarizes the cellular and molecular mechanisms related to the change process of aging. Several of the pathologies associated with aging, such as atherosclerosis and inflammation, involve uncontrolled cellular overgrowth or hyperactivity [2]. The immune system declines in reliability and efficiency with age, resulting in higher risk in the elderly for compromised pathology as a consequence of chronic inflammation such as atherosclerosis, Alzheimer's disease, and an increased susceptibility to infectious disease. Given the complexity of the issue, we attempted to elucidate and categorize the cellular and molecular mechanisms between the inflammasome activation and autophagy that occur with aging. In addition, several studies have indicated that food intake can reduce inflammation activation or increase autophagy to achieve health and longevity. We also discussed the usefulness of natural foods for promoting anti-inflammasome activity.

## 2. The Innate Immune System and Aging

The immune system, which protects an organism from disease, comprises two branches: innate and acquired immunity, including phagocyte lineages, such as macrophages, monocytes, dendritic cells (DCs), neutrophils and natural killer (NK) cells in an innate part, and B and T lymphocytes in an acquired part.  Table 2 summarizes the process of aging-related changes in the immune system.

Innate immunity is the first line of host defense against microbe infection through diverse germline-encoded pattern-recognition receptors (PRRs) in phagocytes, such as Toll-like receptors (TLRs) and nucleotide-binding oligomerization domain- (NOD-) like receptors (NLRs), which recognize pathogen-associated molecular patterns (PAMPs) from pathogens or danger-associated molecular patterns (DAMPs) from damaged tissue in the body [3, 4].

Macrophages, one of the phagocytic cell lineages presented in most tissues, contribute to the innate immunity and are responsible for numerous inflammatory, immunological, and metabolic processes [5]. Macrophages play an important role in the recognition of danger signaling and the initiation of inflammatory responses, including clearance of pathogens via direct phagocytosis of invading pathogens or indirect release of cytokines and chemokines, which can activate and recruit other inflammatory cells to damaged sites. They also activate acquired immunity through the procession of antigens and presentation of peptides to T lymphocytes [5]. Aging-related changes to the macrophages contribute to the aging process via a functional shift toward a proinflammatory phenotype, which constitutively produces more interleukin- (IL-) 6, IL-1beta (IL-1*β*), and tumor necrosis factor (TNF) and reduced phagocytic function [6, 7]. Elevated plasma concentrations of IL-6, IL-1*β*, and TNF have been described in elderly populations and are postulated as predictive markers of functional disability, frailty, and mortality. The macrophage populations of the elderly appear to have reduced levels of major histocompatibility complex (MHC) class II, which contribute to poor CD4^+^ T lymphocyte responses [8]. Furthermore, it has been found that the phagocytic function of macrophages in aging individuals declines. Aging also results in reduced secretion levels of chemokines, such as macrophage inflammatory protein- (MIP-) 1alpha (MIP-1α), MIP-1*β*, MIP-2, and eotaxin [9]. A significant decrease in macrophage precursors and macrophages in the bone marrow of elderly individuals has been described previously [10]. These results suggest that an age-related decline in macrophage function may reduce both innate and adaptive immunities.

Neutrophils are short-lived immune cells that play an important role in the antimicrobial host defense that protects the individual from both bacterial and fungal infections [11]. Phagocytosis, chemotaxis, and ROS production of neutrophils could be changed with aging [12]. Studies measuring phagocytosis of bacteria by the neutrophils have found a significant reduction in phagocytic ability in the elderly population [13, 14]. Larbi et al. [15] demonstrated that the age-related decline in neutrophil functions can be partially explained by the reduced Fc-gamma receptor expression. It has been shown that Fc-gamma receptor-mediated free radical generation and phagocytosis are altered with aging, which is clearly the result of changes in p42/p44 mitogen-activated protein kinase (MAPK) signaling pathways.

DCs play a critical role in linking the innate and the adaptive immune system. DCs are the most potent antigen-presenting cells that can prime naive CD4^+^ T cells via antigen presentation. After Toll-like receptors (TLRs) stimulation such as TLR7 and TLR9, for example, the plasmacytoid dendritic cells (pDCs) produce type I interferon to defend against viral infections and activate NK cells to amplify the host response and help to clear the virus [16–18]. Myeloid dendritic cells (mDCs) are professional antigen-presenting cells to T lymphocytes. They express TLRs and C-type lectin receptors (CLRs) for the detection of viruses. They also produce cytokines, such as IL-12, to induce cytotoxic T-cell responses, which clear virus-infected cells [19]. Epidermal Langerhans cells (LCs), originally described as epidermal DCs, maintain immune homeostasis in the skin by activating skin-resident regulatory T cells. These contain langerin, large granules capable of phagocytosis [20]. LCs are impaired in their phagocytic ability to induce ovalbumin- (OVA-) specific CD4^+^ and CD8^+^ T-cell proliferation in aged mice [20]. Besides presenting the antigens, they also provide the costimulatory signals for optimal activation of NK cells and produce the cytokine IL-17, which is known to recruit neutrophils [21].

## 3. Inflammasome and Aging

In mammals, the inflammasome is a group of cytosolic receptors that recognize not only intracellular PAMPs, but also host-derived signal DAMPs. They control the production of proinflammatory cytokines, such as proinflammatory cytokines IL-1*β* and IL-18. The inflammasome is a multiprotein complex that contains at least two distinct classes of the NLR family or the pyrin domain (PYD) and HIN domain-containing (PYHIN) family. The inflammasome mediates the activation of caspase-1, leading to pro-IL-1*β* and pro-IL-18 processing [22]. In addition to the production of IL-1*β* and IL-18, the inflammasome/caspase-1 complexes may target different effector molecules to regulate diverse physiological functions, such as pyroptosis and tissue repair [23]. During inflammasome activation, NLRP3 can oligomerize through the central nucleotide-binding domain and then recruit an adaptor protein apoptosis-associated speck-like protein containing CARD (ASC) with PYD and an amino-terminal caspase-recruitment-and-activation domain (CARD domain). NLRP3 would interact with the PYD domain of ASC through its own PYD domains, whereas the CARD domain of ASC recruits procaspase-1. Assembly of the inflammasome initiates self-cleavage of caspase-1 and the formation of the active heterotetrameric caspase-1. Active caspase-1 further proteolytically processes pro-IL-1*β* and pro-IL-18 to their mature forms [24]. At least six inflammasome complexes of the NLR family, including NACHT, LRR and PYD domains-containing protein (NLRP)1, NLRP3, CARD domain containing (NLRC)4, NLRP6, NLRP12, and PYHIN family, absent in melanoma 2 (AIM2), have been characterized [4].

Among the numerous inflammasome complexes, the NLRP3 inflammasome has been extensively studied and shown to be activated by a large variety of activators that do not share any structural similarities [3]. The NLRP3 inflammasome is proposed to be activated through a secondary mediator, including potassium efflux, reactive oxygen species (ROS), or lysosomal proteases [4]. The NLRP3 inflammasome requires two signals for its activation in macrophages. Stimulation with lipopolysaccharides (LPS) leads to TLR4 signaling-pathway activation in a nuclear factor kappa-light-chain-enhancer of activated B cells- (NF-*κ*B-) dependent manner. This results in the synthesis of precursor forms of proinflammatory cytokines, including pro-IL-1*β* and NLRP3 proteins [25]. Further stimulation of cells with ATP activates the P_2_X7 receptor, causing K^+^ efflux through membrane pores, which results in the NLRP3 inflammasome assembly. Another proposed mechanism suggests the activation of NLRP3 by cathepsin B released from ruptured lysosomes following the phagocytosis of monosodium urate and alum. It is demonstrated by using cathepsin B inhibitors and lysosome inhibitors* in vitro* [26, 27]. ROS was proposed to be an upstream activator of the NLRP3 inflammasome, originating from the mitochondria (mROS). In contrast, mROS generation from a series of electron transport through the mitochondrial oxidative phosphorylation complex was essential for inflammasome activation. A finding by Zhou et al. [28] reveals that mitochondrial dysfunction activates mROS production. Treatment with NLRP3 activators results in the recruitment of NLRP3 to the mitochondria-associated ER membrane (MAM) where NLRP3 recruited ASC for inflammasome activation. Nakahira et al. [29] also demonstrated that LPS together with ATP causes loss of mitochondrial membrane potential and mROS generation due to the release of mitochondrial DNA (mtDNA). Furthermore, cytosolic mtDNA levels correlate with NLRP3-dependent IL-1*β* production. Interestingly, the findings by Zhou et al. [28] correlated with those by Nakahira et al. [29], which also suggested a role for autophagy in attenuating IL-1*β* production where caspase-1 activation is limited and where the NLRP3 relocates to MAMs through the clearance of damaged mROS production. Other critical effectors of NLRP3 activation have been reported in recent years. Thioredoxin- (TRX-) interacting protein (TXNIP) upon oxidative stress has been shown to dissociate from TRX and bind to NLRP3 to promote the NLRP3 activation and to be linked to the regulation of lipid and glucose metabolism [28]. Micro-RNA-223 and ubiquitination of the NLRP3 are reported as negative regulators of the NLRP3 [30–32]. The NLRP3 inflammasome has been demonstrated to play a critical role in microbial pathogen infection [3, 33]. Nevertheless, dysregulation of the NLRP3 inflammasome activation has been associated with a variety of human diseases, including autoinflammatory diseases, Crohn's disease, type 2 diabetes, atherosclerosis, and cancers [22, 34].

A prominent age-dependent alteration is a slowly progressing proinflammatory phenotype, contributing to a long-term stimulation of the immune system. This can result in a low-grade proinflammatory status referred to as inflammaging, which accompanies the aging process in mammals [35, 36]. Several studies focused on the pattern of transcriptional factors on aging tissues found that overactivation of the I kappa B kinase- (IKK-) NF-*κ*B pathway is one of the signatures of aging, revealing the inflammatory pathways in aging [37, 38]. More evidence shows that systemic inflammation links to inflammaging, including the accumulation of proinflammatory cytokines in metabolic organs, the overexpression of the NF-*κ*B transcription factor in damaged tissue, or a defective autophagy-signaling pathway in phagocytes [36]. Dysregulation of the inflammatory cytokines response, such as IL-1*β*, TNF, and interferon, elicits pathological changes of type 2 diabetes and atherosclerosis, correlated with aging in the human population [22, 34, 39, 40].

## 4. Autophagy and Aging

**Figure 1:**
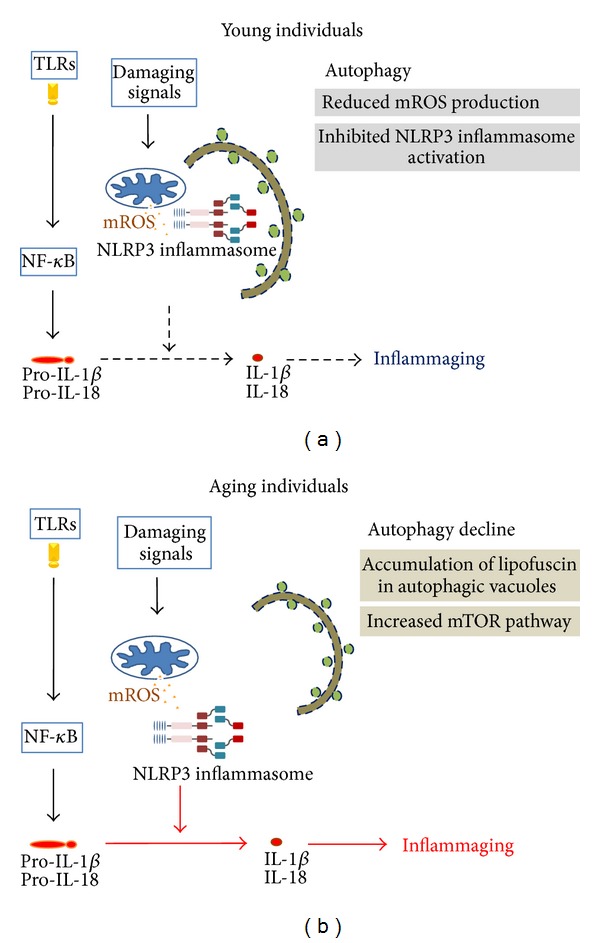
The schematic diagrams represent an overview of the signaling pathways between autophagy and inflammaging. In young individuals (left), autophagy may contribute to maintain the innate physiological lifespan through distinct mechanisms in clearing intracellular mitochondrial ROS (mROS) and NLRP3 inflammasome resulting in decreased inflammaging (black dashed lines), whereas dysfunction of autophagy homeostasis during aging results in increased inflammaging (red lines). However, autophagy protects against the NLRP3 inflammasome-dependent aging process. Aging is only one of the consequences that regulate the depicted signal transduction pathways.

Autophagy is considered an evolutionarily conserved cellular catabolic process, which facilitates the recycling of damaged proteins and organelles [41]. Three distinct types of autophagy coexist in most cells, including macroautophagy (usually referred to as autophagy), microautophagy, and chaperon-mediated autophagy (CMA). The three types of autophagy are well established and carry cytosolic proteins into the lysosomes for degradation. During autophagy, dysfunctional protein or organelles are sequestered into a double-membrane vesicle known as the autophagosome. The origin of the autophagosome, called the phagophore or isolation membrane, may be derived from the plasma membrane, Golgi, mitochondria, and endoplasmic reticulum (ER). Classical autophagy initiation is induced by nutrient deprivation followed by the inhibition of the mammalian target of rapamycin (mTOR), which recruits the UNC-51-like kinase (ULK) complex from the cytosol to the isolation membrane. This leads to the nucleation of the isolation membrane through the assembly of ATG14, Beclin 1, vacuolar protein sorting (VPS)15, class III phosphatidylinositol-3-OH kinase (PI(3)K), and VPS34 complexes. Additional proteins, such as Ambra 1, double FYVE-containing protein (DFCP)1, ATG9, and WD-repeat domain phosphoinositide-interacting (WIPI) protein, also regulate the nucleation step of autophagosome formation [41]. The next step is the elongation of the isolation membrane, which requires two ubiquitin-like protein conjugation systems. The first is the conjugation of ATG12-ATG5, which is covalently linked by ATG7 (E1-like) and ATG10 (E2-like) enzymes, and serves as a dimer complex that associates with ATG16L1. This multiple protein complex is crucial in autophagosome formation. The second is the conjugation of phosphatidylethanolamine- (PE-) microtubule-associated protein 1 light chain 3 (LC3), which is covalently linked by ATG7 (E1-like) and ATG3 (E2-like) enzymes. The ATG5-ATG12–ATG16 complex serves as the E3-like enzyme to generate PE-LC3 (LC3-II), which is incorporated into both the inner and outer membranes of the autophagosome. Autophagy is originally considered to be a nonselective bulk degradation process. Several lines of evidence suggest that selective autophagy occurs through the recognition of autophagy substrates, such as degradation of intracellular bacteria and viruses (xenophagy), regulation of the turnover of mitochondria (mitophagy), the clearance of polyubiquitinated protein aggregates (aggrephagy), and regulation of lipid metabolism (lipophagy). Increasing evidence has revealed that autophagy plays an important role in regulating immune responses and inflammation [41]. The engagement of TLR4 by LPS recruits the Toll-receptor-associated activator of interferon (TRIF) and the myeloid differentiation factor 88 (MyD88) adaptor. This leads to enhanced TRAF6 E3 ligase activity, which results in the K63-linked ubiquitination of Beclin 1 and the oligomerization of Beclin 1. This promotes the activation of PI(3)K and helps the formation of autophagosomes [42, 43]. Another study [44] reported that heat shock protein 90 (HSP90) plays an important role in mediating TLR4-induced autophagy. HSP90 mediates TLR4-induced autophagy through interaction with Beclin 1 and protects Beclin 1 from proteasome-mediated degradation. In addition, HSP90 and the kinase-specific cochaperone Cdc37 interact with ULK1 and promote its stability and activation. This in turn plays an important role in autophagy-mediated mitochondrial clearance [45]. Both TLRs and NLRs can induce autophagy through the activation of Beclin 1. NLRP4, on the other hand, displays an ability to inhibit autophagy [46]. Several studies have reported that autophagy and/or autophagy-related proteins play an important role in regulating mitochondria integrity, ROS generation, and the subsequent NLRP3 activation. Macrophages treated with 3-methyladenine (3MA), a chemical inhibitor of autophagy, or macrophages with the deletion of several autophagic components, including ATG16L1, ATG7, Beclin 1, and LC3, impair mitochondrial homeostasis and further induce more caspase-1 activation and IL-1*β* secretion in response to solely LPS or LPS+NLRP3 agonists [29, 47]. These data strongly suggest that autophagy negatively regulates the NLRP3 inflammasome activity. Autophagy is also a critical regulator of the organelles' homeostasis, particularly for aggregated protein and mitochondria in cells [41]. Damaged mitochondria that have lost their membrane potential and are more likely to release toxic apoptotic mediators and ROS serve as signaling to recruit selective autophagy (mitophagy) [29]. The aging process causes the deficient maintenance of proteostasis (see Table 1), resulting in the accumulation of damaged cellular components in old cells; for example, lipofuscin would destroy lysosome function, thus failing to clear the dysfunctional mitochondria [48]. In particular, dysfunction of mitochondrial homeostasis can increase mROS production and stimulate the NLRP3 inflammasome activation. Thus, autophagy declines with aging, enhancing the inflammaging process (Figure 1). Several regulatory mechanisms indicate that the age-related deficiency of autophagy can enhance the appearance of the inflammation phenotype in cells. It is well known that several redox-sensitive protein kinases, phosphatases, and proinflammatory cytokines can stimulate IKK-NF-*κ*B signaling and ROS production, and the increased levels of ROS can feedback-activate NF-*κ*B signaling. All of them can produce prime activation of the inflammasome. Moreover, declines in autophagy can result in the loss of control activity of the NF-*κ*B complex, which is degraded via selective autophagy [49, 50]. The loss of clearance function of autophagy with aging generates a comfortable situation for stimulating NF-*κ*B signaling directly or indirectly, resulting in inflammasome-dependence in an age-related proinflammatory phenotype manner.

## 5. The Impact of Natural Compounds on Inflammaging

Aging in humans is associated with a high incidence of some chronic diseases and inflammaging-associated diseases, such as type 2 diabetes, atherosclerosis, and Alzheimer's disease. There is a need to develop a preventive strategy for prolonging the period of healthy life and preventing the pathogenesis of these inflammaging-associated diseases. Interestingly, inflammaging-associated diseases are highly related to NLRP3 activation or a decline in autophagy, which increases metabolic and oxidative stress and elevates a low-grade inflammation, which increases the levels of damage.

Food intake including vegetables, fruits, tea, and wine can reduce the development of age-related diseases [51, 52]. Emerging studies suggest that some phytochemical compounds have potential as inflammation inhibitors to impair NLRP3 activation or enhance autophagy. Four main categories of phytochemical compounds may improve inflammaging-related diseases through impaired NLRP3 activation or enhanced autophagy (Table 3).

Resveratrol is a stilbenoid compound that exists in many plant-derived foods such as grapes and red wine [53]. Several discoveries provide evidence demonstrating that a significant amount of resveratrol in the diet has beneficial effects on various chronic diseases and aging. It has been found that resveratrol can protect against type 2 diabetes, heart disease, and Alzheimer's disease [54–56]. In humans, there is still no solid evidence that resveratrol intake can extend lifespan. However, it has been found that resveratrol can protect against NLRP3 inflammasome activation and enhance autophagy [57, 58], which may be able to suppress oxidative stress and inflammation and point to a promising antiaging process.

Other phenolic compounds are described as being linked to anti-inflammatory activity. These compounds include creosol in bamboo vinegar [59] and propolis extracts in Brazilian propolis [60]. They are demonstrated potentially to inhibit NLRP3 activation through the reduction of MAPK and NF-*κ*B activation, decrease ROS production, and impair IL-1*β* expression. All of them are suggested to improve the aging process [52].

Luteoloside and quercetin, naturally occurring flavonoids in food, exhibit health-beneficial properties and an antiaging effect for humans [52]. Luteoloside, isolated from the medicinal plant* Gentiana macrophylla*, has been demonstrated to show an anticancer effect against hepatocellular carcinoma (HCC) cells through its effect of inhibiting the NLRP3 inflammasome through inhibiting proliferation, invasion, and metastasis of HCC cells in a mouse lung metastasis model [61]. Quercetin isolated from herbal foods has been reported previously to exhibit potential for anti-inflammation and antihyperlipidemia [62, 63]. Recent studies [64, 65] have demonstrated that quercetin could impair NLRP3 inflammasome activation to improve renal inflammation.

Catechins and epigallocatechin-3-gallate (EGCG) are abundant in teas derived from the tea plant* Camellia sinensis*. These products show the effect of ameliorating a variety of human diseases such as cancers, atherosclerotic lesions, and Alzheimer's disease [66–70]. Recent studies [37, 38] have shown that they also attenuate the* Helicobacter pylori*-triggered caspase-1 signaling pathway, oxidative stress, and apoptosis in the gastric mucosa of the* Helicobacter pylori*-infected mouse model.

## 6. Conclusion

A healthy lifestyle to avoid premature aging is achievable through maintaining a happy, relaxed mood, engaging in regular sports and exercise, not smoking or drinking, and following a nutrient-rich, low-calorie diet. Studies have shown that people who do not have a healthy lifestyle and do not adhere to a nutritious diet are at high risk of age-related diseases such as type 2 diabetes, cancer, and cardiovascular disease. In this review, we summarize the relationship between inflammaging and autophagy. There are indications that autophagic capacity is dysfunctional in age-related diseases. Autophagy declines with aging, triggering NLRP3 activation, and enhancing the inflammaging process. Decreased NLRP3 activation and increased autophagy can extend the lifespan. In this respect, the effective function of autophagic uptake in the clearance of dysfunctional mitochondria reduced oxidative stress and impaired NLRP3 activation is critical to maintaining cell homeostasis. Growing evidence shows that some foods containing natural compounds, such as resveratrol, catechins, EGCG, propolis extracts, creosol, and luteoloside, are categorized as antiaging molecules [52]. There is suggestion that dietary intake of these compounds may promote health and extend the lifespan via multiple mechanisms, including the reduction of oxidative stress, induction of autophagy, and suppression of NLRP3 activation. This can lead to a healthier and longer lifespan.

## Figures and Tables

**Table 1:** The impact of aging on lifespans.

The molecular hallmarks of age	Age-related changes	Reference
Genomic instability		
Nuclear DNA	Chromosomal aneuploidies and copy number variations	[71–73]
Mitochondrial DNA	Replication errors cause polyclonal expansion of mtDNA mutations	[74]
Telomere exhaustion	Shortened telomeres exhibit decreased lifespans	[75, 76]
Epigenetic alterations		
Histone modifications	Deficiency in SIRT6 exhibits accelerated aging	[77]
DNA methylation	Polycomb target genes become hypermethylated with age	[78]
Chromatin remodeling	HP1α effects longevity in flies	[79]
Transcriptional alterations	Micro-RNA mir-71 is required for the lifespan extension	[80]
Loss of proteostasis		
Chaperone-mediated protein folding and stability	HSPs decline on longevity Accumulation and aggregation of abnormal proteins occur in aged organism	[81, 82]
Delay or dysfunction of autophagy	mTOR signaling in the regulation of mammalian lifespan	[83]
The ubiquitin-proteasome system	Enhancement of proteasome activity extends replicative lifespan in yeast	[84]
Deregulated nutrient sensing		
The insulin- and IGF-1-signaling pathway	Levels decline and dysfunction of GH/IGF-1 signaling pathway	[39, 85]
mTOR and AMPK	Inhibition of mTOR/DR pathway extends lifespan	[86, 87]
Mitochondrial dysfunction		
ROS	Amphibious effects of ROS on aging	[88–91]
Mitochondrial Integrity and Biogenesis	Reduced efficiency of telomerase activation with aging	[92]
Cellular senescence		
The INK4a/ARF Locus	Ink4a/ARF expression increases aging	[93, 94]
Stem cell attrition	Hematopoiesis declines with age resulting in a diminished production of adaptive immune cells Reduced in cell-cycle activity of hematopoietic stem cells (HSCs) on aged mice	[7, 95]
Inflammation	Activation of the NLRP3 inflammasome leading to increased production of IL-1*β*, TNF, and interferons	[36, 96]

SIRT6: sirtuin-6; HP1α: heterochromatin protein 1α; HSPs: heat shock proteins; mTOR: mammalian target of rapamycin; IGF-1: insulin/insulin growth factor 1; DR: dietary restriction; ROS: reactive oxygen species; AMPK: AMP-activated protein kinase; HSCs: hematopoietic stem cells; NLRP3: nucleotide-binding domain, leucine rich family (NLR), pyrin containing 3; IL-1*β*: interleukin-1*β*; TNF: tumor necrosis factor.

**Table 2:** Alterations in the immune system associated with aging.

Immune system	Age-related changes	References
Innate immunity	** **	** **
Monocytes or macrophages	Reduced levels of MHC class II complexes, reduced phagocytic capacity, and enhanced oxidative stress	[97, 98]
Neutrophils	Reduction in phagocytosis ability, impaired free radical production, and decreased rescue from apoptosis	[99]
Dendritic cells	Reduced antigen presentation and impaired phagocytic capability to clean apoptotic cells	[100]
Natural killer cells	Increased number of NK cells, reduced cytotoxicity, and impaired proliferation ability in response to IL-2 stimulation	[98]
Acquired immunity	** **	** **
T cells	Reductions in T-cell thymopoiesis, accumulated highly differentiated memory T cells, loss of CD28 antigen and CD69 antigen for T cell activation and signal transduction, and reduced CD8+ cell proliferation in response to antigen stimulation	[101, 102]
B cells	Reductions in B-cell lymphopoiesis, increased memory B cells and fewer naive B cells, impaired antibody response to vaccination, and increased production of low-affinity antibodies due to decreased isotype switching	[98]

MHC II: major histocompatibility complex class II.

**Table 3:** Classification of compounds from food sources associated with anti-NLRP3 inflammasome.

Category	Compounds	Molecular mechanism		Resources	References
Stilbenoids	Resveratrol	Inhibited NLRP3 activation Induced autophagy	Impaired caspase-1 and IL-1*β* expression Reduced the acetylation of cytoplasmic proteins	*Veratrum album *	[57, 58]
Flavonoids					
Flavonols	Quercetin	Suppressed renal NLRP3 activation	Impaired caspase-1 and IL-1*β* expression	Quercetum	[64, 65]
Flavones	Luteoloside	Inhibited NLRP3 activation	Reduced ROS accumulation Impaired NLRP3, caspase-1, and IL-1*β* expression	Honeysuckle	[61]
Flavan-3-ols	Catechins	Inhibited NLRP3 activation Enhanced autophagy	Impaired caspase-1 and IL-1*β* expression Enhanced Beclin 1 expression	Green tea	[37, 38]
	EGCG	Inhibited NLRP3 activation Enhanced autophagy	Reduced ROS accumulation, NF-*κ*B activation, and NLRP3 expression Impaired caspase-1 and IL-1*β* expression	Green tea	[103–105]
Other phenolic compounds	Creosol	Impaired NLRP3 activation	Reduced iNOS expression and NO levels Decreased ROS production Impaired IL-1*β* expression	Bamboo vinegar (BV)	[59]
	Propolis extracts	Inhibited NLRP3 activation	Reduced the IL-1*β* secretion	Brazilian propolis	[60]

iNOS: inducible nitric oxide synthase; ROS: reactive oxygen species; IL: interleukin; MAPK: mitogen-activated protein kinase; EGCG: epigallocatechin-3-gallate; NF-*κ*B: nuclear factor kappa-light-chain-enhancer of activated B cells.

## References

[1] Larbi A, Rymkiewicz P, Vasudev A (2013). The immune system in the elderly: a fair fight against diseases?. *Aging Health*.

[2] Blagosklonny MV (2008). Aging: ROS or TOR. *Cell Cycle*.

[3] Davis BK, Wen H, Ting JP-Y (2011). The Inflammasome NLRs in immunity, inflammation, and associated diseases. *Annual Review of Immunology*.

[4] Rathinam VAK, Vanaja SK, Fitzgerald KA (2012). Regulation of inflammasome signaling. *Nature Immunology*.

[5] Chow A, Brown BD, Merad M (2011). Studying the mononuclear phagocyte system in the molecular age. *Nature Reviews Immunology*.

[6] Sadeghi HM, Schnelle JF, Thomas JK, Nishanian P, Fahey JL (1999). Phenotypic and functional characteristics of circulating monocytes of elderly persons. *Experimental Gerontology*.

[7] Shaw AC, Joshi S, Greenwood H, Panda A, Lord JM (2010). Aging of the innate immune system. *Current Opinion in Immunology*.

[8] Wang CQ, Udupa KB, Xiao H, Lipschitz DA (1995). Effect of age on marrow macrophage number and function. *Aging*.

[9] Swift ME, Burns AL, Gray KL, DiPietro LA (2001). Age-related alterations in the inflammatory response to dermal injury. *Journal of Investigative Dermatology*.

[10] Ogawa T, Kitagawa M, Hirokawa K (2000). Age-related changes of human bone marrow: a histometric estimation of proliferative cells, apoptotic cells, T cells, B cells and macrophages. *Mechanisms of Ageing and Development*.

[11] Mócsai A (2013). Diverse novel functions of neutrophils in immunity, infammation, and beyond. *Journal of Experimental Medicine*.

[12] Shaw AC, Goldstein DR, Montgomery RR (2013). Age-dependent dysregulation of innate immunity. *Nature Reviews Immunology*.

[13] Khanfer R, Carroll D, Lord JM, Phillips AC (2012). Reduced neutrophil superoxide production among healthy older adults in response to acute psychological stress. *International Journal of Psychophysiology*.

[14] Wenisch C, Patruta S, Daxböck F, Krause R, Hörl W (2000). Effect of age on human neutrophil function. *Journal of Leukocyte Biology*.

[15] Larbi A, Douziech N, Fortin C, Linteau A, Dupuis G, Fulop T (2005). The role of the MAPK pathway alterations in GM-CSF modulated human neutrophil apoptosis with aging. *Immunity and Ageing*.

[16] Agrawal A, Gupta S (2011). Impact of aging on dendritic cell functions in humans. *Ageing Research Reviews*.

[17] Romagnani C, Della Chiesa M, Kohler S (2005). Activation of human NK cells by plasmacytoid dendritic cells and its modulation by CD4+ T helper cells and CD4+ CD25hi T regulatory cells. *European Journal of Immunology*.

[18] Lande R, Gilliet M (2010). Plasmacytoid dendritic cells: key players in the initiation and regulation of immune responses. *Annals of the New York Academy of Sciences*.

[19] Kawai T, Akira S (2007). Signaling to NF-*κ*B by Toll-like receptors. *Trends in Molecular Medicine*.

[20] Seneschal J, Clark RA, Gehad A, Baecher-Allan CM, Kupper TS (2012). Human epidermal Langerhans cells maintain immune homeostasis in skin by activating skin resident regulatory T cells. *Immunity*.

[21] Stout-Delgado HW, Du W, Shirali AC, Booth CJ, Goldstein DR (2009). Aging promotes neutrophil-induced mortality by augmenting IL-17 production during viral infection. *Cell Host and Microbe*.

[22] Zitvogel L, Kepp O, Galluzzi L, Kroemer G (2012). Inflammasomes in carcinogenesis and anticancer immune responses. *Nature Immunology*.

[23] Miao EA, Rajan JV, Aderem A (2011). Caspase-1-induced pyroptotic cell death. *Immunological Reviews*.

[24] Latz E, Xiao TS, Stutz A (2013). Activation and regulation of the inflammasomes. *Nature Reviews Immunology*.

[25] Guarda G, Zenger M, Yazdi AS (2011). Differential expression of NLRP3 among hematopoietic cells. *Journal of Immunology*.

[26] Martinon F, Pétrilli V, Mayor A, Tardivel A, Tschopp J (2006). Gout-associated uric acid crystals activate the NALP3 inflammasome. *Nature*.

[27] Hornung V, Bauernfeind F, Halle A (2008). Silica crystals and aluminum salts activate the NALP3 inflammasome through phagosomal destabilization. *Nature Immunology*.

[28] Zhou R, Tardivel A, Thorens B, Choi I, Tschopp J (2010). Thioredoxin-interacting protein links oxidative stress to inflammasome activation. *Nature Immunology*.

[29] Nakahira K, Haspel JA, Rathinam VAK (2011). Autophagy proteins regulate innate immune responses by inhibiting the release of mitochondrial DNA mediated by the NALP3 inflammasome. *Nature Immunology*.

[30] Bauernfeind F, Rieger A, Schildberg FA, Knolle PA, Schmid-Burgk JL, Hornung V (2012). NLRP3 inflammasome activity is negatively controlled by miR-223. *Journal of Immunology*.

[31] Haneklaus M, Gerlic M, Kurowska-Stolarska M (2012). Cutting edge: miR-223 and EBV miR-BART15 regulate the NLRP3 inflammasome and IL-1*β* production. *The Journal of Immunology*.

[32] Juliana C, Fernandes-Alnemri T, Kang S, Farias A, Qin F, Alnemri ES (2012). Non-transcriptional priming and deubiquitination regulate NLRP3 inflammasome activation. *Journal of Biological Chemistry*.

[33] Franchi L, Muñoz-Planillo R, Núñez G (2012). Sensing and reacting to microbes through the inflammasomes. *Nature Immunology*.

[34] Yang C-S, Shin D-M, Jo E-K (2012). The role of NLR-related protein 3 inflammasome in host defense and inflammatory diseases. *International Neurourology Journal*.

[35] Franceschi C, Bonafè M, Valensin S (2000). Inflamm-aging: an evolutionary perspective on immunosenescence. *Annals of the New York Academy of Sciences*.

[36] Salminen A, Kaarniranta K, Kauppinen A (2012). Inflammaging: disturbed interplay between autophagy and inflammasomes. *Aging*.

[37] Yang J-C, Yang H-C, Shun C-T, Wang T-H, Chien C-T, Kao JY (2013). Catechins and sialic acid attenuate helicobacter pylori -triggered epithelial caspase-1 activity and eradicate helicobacter pylori infection. *Evidence-Based Complementary and Alternative Medicine*.

[38] Yang JC, Shun CT, Chien CT, Wang TH (2008). Effective prevention and treatment of Helicobacter pylori infection using a combination of catechins and sialic acid in AGS cells and BALB/c mice. *Journal of Nutrition*.

[39] Barzilai N, Huffman DM, Muzumdar RH, Bartke A (2012). The critical role of metabolic pathways in aging. *Diabetes*.

[40] Tabas I (2010). Macrophage death and defective inflammation resolution in atherosclerosis. *Nature Reviews Immunology*.

[41] Levine B, Mizushima N, Virgin HW (2011). Autophagy in immunity and inflammation. *Nature*.

[42] Xu Y, Jagannath C, Liu XD, Sharafkhaneh A, Kolodziejska KE, Eissa NT (2007). Toll-like receptor 4 is a sensor for autophagy associated with innate immunity. *Immunity*.

[43] Shi C-S, Kehrl JH (2010). Traf6 and A20 differentially regulate TLR4-induced autophagy by affecting the ubiquitination of Beclin 1. *Autophagy*.

[44] Xu C, Liu J, Hsu L-C, Luo Y, Xiang R, Chuang T-H (2011). Functional interaction of heat shock protein 90 and Beclin 1 modulates Toll-like receptor-mediated autophagy. *The FASEB Journal*.

[45] Joo JH, Dorsey FC, Joshi A (2011). Hsp90-Cdc37 chaperone complex regulates Ulk1- and Atg13-mediated mitophagy. *Molecular Cell*.

[46] Jounai N, Kobiyama K, Shiina M, Ogata K, Ishii KJ, Takeshita F (2011). NLRP4 negatively regulates autophagic processes through an association with Beclin1. *The Journal of Immunology*.

[47] Saitoh T, Fujita N, Jang MH (2008). Loss of the autophagy protein Atg16L1 enhances endotoxin-induced IL-1*β* production. *Nature*.

[48] Terman A, Kurz T, Navratil M, Arriaga EA, Brunk UT (2010). Mitochondrial Turnover and aging of long-lived postmitotic cells: The mitochondrial-lysosomal axis theory of aging. *Antioxidants and Redox Signaling*.

[49] Chang CP, Su YC, Hu CW, Lei HY (2013). TLR2-dependent selective autophagy regulates NF-*κ*B lysosomal degradation in hepatoma-derived M2 macrophage differentiation. *Cell Death and Differentiation*.

[50] Paul S, Kashyap AK, Jia W, He Y-W, Schaefer BC (2012). Selective autophagy of the adaptor protein Bcl10 modulates T cell receptor activation of NF-kappaB. *Immunity*.

[51] Everitt AV, Hilmer SN, Brand-Miller JC (2006). Dietary approaches that delay age-related diseases. *Clinical Interventions in Aging*.

[52] Si H, Liu D (2014). Dietary antiaging phytochemicals and mechanisms associated with prolonged survival. *The Journal of Nutritional Biochemistry*.

[53] Gu X, Creasy L, Kester A, Zeece M (1999). Capillary electrophoretic determination of resveratrol in wines. *Journal of Agricultural and Food Chemistry*.

[54] Frankel EN, Waterhouse AL, Kinsella JE (1993). Inhibition of human LDL oxidation by resveratrol. *The Lancet*.

[55] Smoliga JM, Baur JA, Hausenblas HA (2011). Resveratrol and health—a comprehensive review of human clinical trials. *Molecular Nutrition and Food Research*.

[56] Baur JA, Sinclair DA (2006). Therapeutic potential of resveratrol: the in vivo evidence. *Nature Reviews Drug Discovery*.

[57] Yang SJ, Lim Y (2014). Resveratrol ameliorates hepatic metaflammation and inhibits NLRP3 inflammasome activation. *Metabolism-Clinical and Experimenta*.

[58] Pietrocola F, Mariño G, Lissa D (2012). Pro-autophagic polyphenols reduce the acetylation of cytoplasmic proteins. *Cell Cycle*.

[59] Ho CL, Lin CY, Ka SM (2013). amboo vinegar decreases inflammatory mediator expression and NLRP3 inflammasome activation by inhibiting reactive oxygen species generation and protein kinase C-alpha/delta activation. *PLoS ONE*.

[60] Hori JI, Zamboni DS, Carrão DB, Goldman GH, Berretta AA (2013). The inhibition of inflammasome by Brazilian propolis (EPP-AF). *Evidence-Based Complementary and Alternative Medicine*.

[61] Fan SH, Wang YY, Lu J (2014). Luteoloside suppresses proliferation and metastasis of hepatocellular carcinoma cells by inhibition of NLRP3 inflammasome. *PLoS ONE*.

[62] Egert S, Bosy-Westphal A, Seiberl J (2009). Quercetin reduces systolic blood pressure and plasma oxidised low-density lipoprotein concentrations in overweight subjects with a high-cardiovascular disease risk phenotype: a double-blinded, placebo-controlled cross-over study. *The British Journal of Nutrition*.

[63] Zhu JX, Wang Y, Kong LD, Yang C, Zhang X (2004). Effects of Biota orientalis extract and its flavonoid constituents, quercetin and rutin on serum uric acid levels in oxonate-induced mice and xanthine dehydrogenase and xanthine oxidase activities in mouse liver. *Journal of Ethnopharmacology*.

[64] Wang C, Pan Y, Zhang Q-Y, Wang F-M, Kong L-D (2012). Quercetin and allopurinol ameliorate kidney injury in STZ-treated rats with regulation of renal NLRP3 inflammasome activation and lipid accumulation. *PLoS ONE*.

[65] Hu Q-H, Zhang X, Pan Y, Li Y-C, Kong L-D (2012). Allopurinol, quercetin and rutin ameliorate renal NLRP3 inflammasome activation and lipid accumulation in fructose-fed rats. *Biochemical Pharmacology*.

[66] Arts ICW, Jacobs DR, Harnack LJ, Gross M, Folsom AR (2001). Dietary catechins in relation to coronary heart disease death among postmenopausal women. *Epidemiology*.

[67] Babu PVA, Liu D (2008). Green tea catechins and cardiovascular health: an update. *Current Medicinal Chemistry*.

[68] Higdon JV, Frei B (2003). Tea catechins and polyphenols: health effects, metabolism, and antioxidant functions. *Critical Reviews in Food Science and Nutrition*.

[69] Shankar S, Ganapathy S, Hingorani SR, Srivastava RK (2008). EGCG inhibits growth, invasion, angiogenesis and metastasis of pancreatic cancer. *Frontiers in Bioscience*.

[70] Haque AM, Hashimoto M, Katakura M, Tanabe Y, Hara Y, Shido O (2006). Long-term administration of green tea catechins improves spatial cognition learning ability in rats. *Journal of Nutrition*.

[71] López-Otín C, Blasco MA, Partridge L, Serrano M, Kroemer G (2013). The hallmarks of aging. *Cell*.

[72] Forsberg LA, Rasi C, Razzaghian HR (2012). Age-related somatic structural changes in the nuclear genome of human blood cells. *The American Journal of Human Genetics*.

[73] Faggioli F, Wang T, Vijg J, Montagna C (2012). Chromosome-specific accumulation of aneuploidy in the aging mouse brain. *Human Molecular Genetics*.

[74] Payne BAI, Wilson IJ, Hateley CA (2011). Mitochondrial aging is accelerated by anti-retroviral therapy through the clonal expansion of mtDNA mutations. *Nature Genetics*.

[75] Jaskelioff M, Muller FL, Paik JH (2011). Telomerase reactivation reverses tissue degeneration in aged telomerase-deficient mice. *Nature*.

[76] Boonekamp JJ, Simons MJP, Hemerik L, Verhulst S (2013). Telomere length behaves as biomarker of somatic redundancy rather than biological age. *Aging Cell*.

[77] Mostoslavsky R, Chua KF, Lombard DB (2006). Genomic instability and aging-like phenotype in the absence of mammalian SIRT6. *Cell*.

[78] Maegawa S, Hinkal G, Kim HS (2010). Widespread and tissue specific age-related DNA methylation changes in mice. *Genome Research*.

[79] Larson K, Yan S-J, Tsurumi A (2012). Heterochromatin formation promotes longevity and represses ribosomal RNA synthesis. *PLoS Genetics*.

[80] Boulias K, Horvitz HR (2012). The C. elegans MicroRNA mir-71 acts in neurons to promote germline-mediated longevity through regulation of DAF-16/FOXO. *Cell Metabolism*.

[81] Walker GA, Lithgow GJ (2003). Lifespan extension in *C. elegans* by a molecular chaperone dependent upon insulin-like signals. *Aging Cell*.

[82] Koga H, Kaushik S, Cuervo AM (2011). Protein homeostasis and aging: the importance of exquisite quality control. *Ageing Research Reviews*.

[83] Harrison DE, Strong R, Sharp ZD (2009). Rapamycin fed late in life extends lifespan in genetically heterogeneous mice. *Nature*.

[84] Kruegel U, Robison B, Dange T (2011). Elevated proteasome capacity extends replicative lifespan in saccharomyces cerevisiae. *PLoS Genetics*.

[85] Schumacher B, van der Pluijm I, Moorhouse MJ (2008). Delayed and accelerated aging share common longevity assurance mechanisms. *PLoS Genetics*.

[86] Johnson SC, Rabinovitch PS, Kaeberlein M (2013). MTOR is a key modulator of ageing and age-related disease. *Nature*.

[87] Mair W, Morantte I, Rodrigues APC (2011). Lifespan extension induced by AMPK and calcineurin is mediated by CRTC-1 and CREB. *Nature*.

[88] Harman D (1965). The free radical theory of aging: effect of age on serum copper levels. *Journal of Gerontology*.

[89] Hekimi S, Lapointe J, Wen Y (2011). Taking a “good” look at free radicals in the aging process. *Trends in Cell Biology*.

[90] van Remmen H, Ikeno Y, Hamilton M (2003). Life-long reduction in MnSOD activity results in increased DNA damage and higher incidence of cancer but does not accelerate aging. *Physiological Genomics*.

[91] Ristow M, Schmeisser S (2011). Extending life span by increasing oxidative stress. *Free Radical Biology and Medicine*.

[92] Sahin E, DePinho RA (2012). Axis of ageing: telomeres, p53 and mitochondria. *Nature Reviews—Molecular Cell Biology*.

[93] Krishnamurthy J, Torrice C, Ramsey MR (2004). Ink4a/Arf expression is a biomarker of aging. *Journal of Clinical Investigation*.

[94] Ressler S, Bartkova J, Niederegger H (2006). p16INK4A is a robust in vivo biomarker of cellular aging in human skin. *Aging Cell*.

[95] Rossi DJ, Bryder D, Seita J, Nussenzweig A, Hoeijmakers J, Weissman IL (2007). Deficiencies in DNA damage repair limit the function of haematopoietic stem cells with age. *Nature*.

[96] Green DR, Galluzzi L, Kroemer G (2011). Mitochondria and the autophagy-inflammation-cell death axis in organismal aging. *Science*.

[97] Plowden J, Renshaw-Hoelscher M, Engleman C, Katz J, Sambhara S (2004). Innate immunity in aging: impact on macrophage function. *Aging Cell*.

[98] Weiskopf D, Weinberger B, Grubeck-Loebenstein B (2009). The aging of the immune system. *Transplant International*.

[99] Fulop T, le Page A, Fortin C (2014). Cellular signaling in the aging immune system. *Current Opinion in Immunology*.

[100] Agrawal A, Agrawal S, Gupta S (2007). Dendritic cells in human aging. *Experimental Gerontology*.

[101] Serra JA, Fernandez-Gutierrez B, Hernandez-Garcia C (1996). Early T cell activation in elderly humans. *Age and Ageing*.

[102] Baylis D, Bartlett DB, Patel HP, Roberts HC (2013). Understanding how we age: insights into inflammaging. *Longevity and Healthspan*.

[103] Ellis LZ, Liu W, Luo Y (2011). Green tea polyphenol epigallocatechin-3-gallate suppresses melanoma growth by inhibiting inflammasome and IL-1*β* secretion. *Biochemical and Biophysical Research Communications*.

[104] Tsai P-Y, Ka S-M, Chang J-M (2011). Epigallocatechin-3-gallate prevents lupus nephritis development in mice via enhancing the Nrf2 antioxidant pathway and inhibiting NLRP3 inflammasome activation. *Free Radical Biology and Medicine*.

[105] Li W, Zhu S, Li J (2011). EGCG stimulates autophagy and reduces cytoplasmic HMGB1 levels in endotoxin-stimulated macrophages. *Biochemical Pharmacology*.

